# The Cobra sign: A marker for overestimation of tendon retraction in proximal semimembranosus tendon avulsion

**DOI:** 10.1002/ksa.12437

**Published:** 2024-08-22

**Authors:** Nicolas Lefèvre, Mohamad K. Moussa, Ahmad Chahal, Alain Meyer, Olivier Grimaud, Zeinab M. Khalaf, Ali Alayane, Yoann Bohu, Alexandre Hardy

**Affiliations:** ^1^ Clinique du Sport Paris France; ^2^ Department of Orthopedic Surgery Group Hospitalier Sélestat Obernai Séléstat Alsace France; ^3^ Department of Radiology Hôpital Saint Camille Bry‐sur‐Marne France

**Keywords:** hamstring avulsion, MRI retraction, MRI sign, semimembranosus, surgical retraction

## Abstract

**Purpose:**

To introduce a new magnetic resonance imaging (MRI) sign, termed the Cobra sign, and identify its diagnostic metrics. The secondary aim was to demonstrate that this sign can be a source of false evaluation of tendon retraction in patients with proximal hamstring avulsion injury.

**Method:**

This retrospective cohort study targeted patients surgically treated for proximal hamstring avulsion injury from January 2019 to June 2023. The MRI Cobra sign was defined as a wavy curved T2‐hypointense band with the free end folding distally over itself, resembling a cobra head. The primary outcome measure was the characterization of the Cobra sign in patients with proximal hamstring avulsion injury. The secondary outcome was the association of this sign with tendon retraction. The study included 81 proximal hamstring avulsion injury patients (mean age of 45.7, SD = 13.9), with 41 (50.6%) complete avulsions, 33 semimembranosus, and 7 conjoint tendons.

**Results:**

The MRI Cobra sign was found in 25 patients (17 semimembranosus and 8 complete). It was confirmed surgically only in semimembranosus cases. It demonstrated 51.5% sensitivity and 83.3% specificity for isolated semimembranosus avulsions, with a significant positive likelihood ratio of 3.0. MRI retraction was 10.05 cm (±3.0), reducing to 7.9 cm (±2.5) on surgical measurement (mean difference = 2.0 cm, *p* < 0.001). The regression analysis confirmed MRI retraction's influence on the Cobra sign, with a 1.4 odds increase per unit (*p* < 0.001). In linear regression analysis, each unit increase in MRI retraction corresponded to a 79% increase in surgical retraction (coefficient 0.7, *t* = 11.1, *p* < 0.001).

**Conclusion:**

The Cobra sign demonstrated acceptable diagnostic accuracy for isolated semimembranosus avulsion, with a high specificity of 83.3%, a low sensitivity of 51.5%, and a positive likelihood ratio of 3.0. The presence of the Cobra sign indicates an overestimated MRI retraction by approximately 21%.

**Level of Evidence:**

Level III.

**Abbreviations**
DICOM: digital imaging and communications in medicine
MRI: magnetic resonance imaging
SM: semimembranosus

## INTRODUCTION

Proximal hamstring avulsion injury is a rare athletic injury that occurs as a result of overstretching or high‐speed running [2, 19, 20, 21]. Different mechanisms can lead to different types of injury, making this entity a heterogeneous group regarding tendon involvement, the injury location, and the size of the injury [2, 3, 20]. High‐speed injuries are thought to be associated with biceps femoris myotendinous injury, whereas overstretching injuries are associated with semimembranosus (SM) rupture [2, 3, 4]. It has been shown to be associated with decreased sports levels and can result in significant functional impairment if not treated appropriately [18, 21].

Imaging plays an important role in the diagnosis, treatment orientation, and prognosis assessment of proximal hamstring avulsion injury [15, 25, 26]. Magnetic resonance imaging (MRI) is considered the gold standard imaging modality to confirm the diagnosis and evaluate the extent of injury [11, 17]. It also allows for the assessment of the degree of tendon retraction, its relation to the sciatic nerve, and the morphological features of the free tendon end, all of which are important elements to consider in treatment planning [15, 17]. Retraction assessment is one of the key information in the assessment of proximal hamstring avulsion injury as studies show that complete injuries with retraction had worse outcomes compared to those without retraction and recommended surgical intervention [26]. A recent systematic review by Jokela et al. found that there is variability and weakness in the current literature on the determination of tendon retraction in cases of proximal hamstring avulsion injury [13]. Six et al. reported moderate agreement in quantifying retraction and difficulty in locating the retracted tendon stump, reported by almost half of the radiologists [28]. Bloom et al. noted significant variability in reporting and measurement of retraction among musculoskeletal radiologists and orthopaedic surgeons, partially due to the wide surface area of the ischial tuberosity (10 cm²), allowing for greater potential variability in measurements [8]. Accurate quantification of retraction is crucial on three fronts: it aids in surgical decision‐making [9, 20], determines the need for graft augmentation in case of chronic injuries [22, 24], and assesses the reparability of the tendon [7].

The aim of this study was to introduce a new MRI sign, termed the Cobra sign, resulting from the folding of the free end of the retracted proximal hamstring avulsion injury tendon over itself and identify its diagnostic metrics. The secondary aim was to demonstrate that this sign can be a source of false evaluation of tendon retraction in patients with proximal hamstring avulsion injury.

The study hypothesis was that this sign is specifically associated with isolated SM rupture. The secondary hypothesis was that measuring the tendon retraction to the folding angle of the tendon over itself would lead to overestimated retraction.

## MATERIALS AND METHODS

This is a retrospective analysis of prospectively collected data conducted at a sports surgery referral centre, targeting patients surgically treated for proximal hamstring avulsion injury between January 2023 and June 2023. Due to its rarity [12, 13], the timeframe for inclusion of SM tendon avulsion was extended back to January 2019. The study was approved by the Ethics Committee of the Pitié Salpétrière University Hospital (ID CPP‐IDF6‐19012012), and informed consent was obtained.

Inclusion criteria were patients with a surgically confirmed diagnosis of proximal hamstring avulsion injury enrolled in the PHAS Cohort study (Proximal Hamstring Avulsion Cohort Study, NCT number: NCT02906865). Exclusion criteria included patients with missing MRIs, those who had revision surgery, those with bony avulsion, and those who refused enrollment in the study.

Partial injuries were defined as the avulsion of SM alone or the conjoint tendon (semitendinosus and biceps) alone. Complete injuries were defined as avulsion of all tendons [12, 13]. Surgery was offered to patients with complete injuries, those with partial injuries exhibiting more than 2 cm of retraction, patients with partial injuries who did not respond to 6 months of conservative treatment, and professional/competitive athletes with partial injuries [5, 9, 12, 21, 25, 27].

### MRI protocol

The study utilized two sources of MRI data. For patients referred to the centre with a confirmed diagnosis of proximal hamstring avulsion injury from another medical centre, the original MRI data were used and recorded as DICOM files. For patients referred to the centre with a suspected proximal hamstring avulsion injury, an MRI was ordered and performed at the centre using a GE Healthcare Signa 3 Tesla Architect MRI machine. The analysed sequences in these MRI images were mainly axial and coronal T2‐weighted sequences. The MRI assessment was performed by one expert orthopaedic surgeon (Nicolas Lefèvre) and one independent musculoskeletal radiologist (Ahmad Chahal).

### MRI retraction

MRI retraction was measured as the distance between the hamstring footprint and the most proximal part of the tendon stump [24].

**Figure 1:**
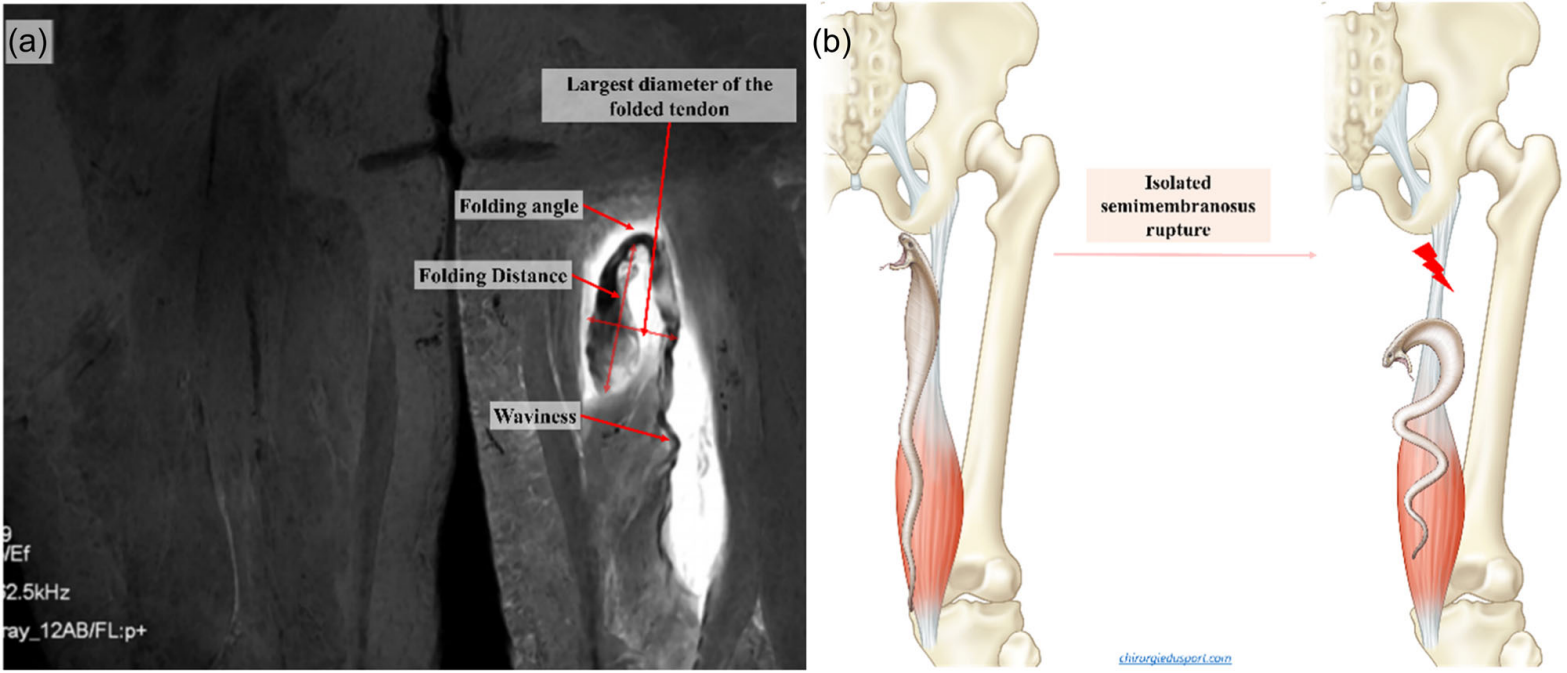
(a) A coronal T2‐weighted magnetic resonance imaging (MRI) showing an isolated semimembranosus rupture with an appearance resembling the Cobra head. (b) An illustration demonstrating the theoretical mechanism of the Cobra sign: the cobra represents the semimembranosus tendon on the left, attached to the ischial tuberosity. On the right, in the case of a proximal tendon rupture, the cobra will fold over itself.

In line with the study's hypothesis, MRI evaluation focused on the identification of areas where the proximal stump of the proximal hamstring tendons forms a region where the tendon folds over itself, termed the Cobra sign. This sign was specifically referred to as a wavy curved T2‐hypointense band with the free end folding distally on the tendon, resembling a cobra head and creating a ‘false stump’ at the ‘folding angle’ (Figure 1a,b).

Among patients exhibiting the Cobra sign, specific measurements were quantified:

– The presence of tendon waviness; this term was employed by van der Made et al. [30] to refer to the sinusoidal curvature that can exhibit the injured hamstring tendon in the intramuscular region.
– The ‘folding distance’ which was defined by the study as the measurement from the folding angle to the end of the folded tendon.
– The ‘largest folding diameter' defined as the diameter of the tendon at the site where it is doubled over itself.

### Surgical retraction

Before any release, tendon retraction was measured once the tendon stump was localized within its fibrotic tissue. This was done using a metallic ruler marked in centimetres, allowing for measurements to the nearest millimetre to ensure accuracy.

The distance between the hamstring footprint and the proximal stump was referred to as the apparent surgical retraction.

**Figure 2:**
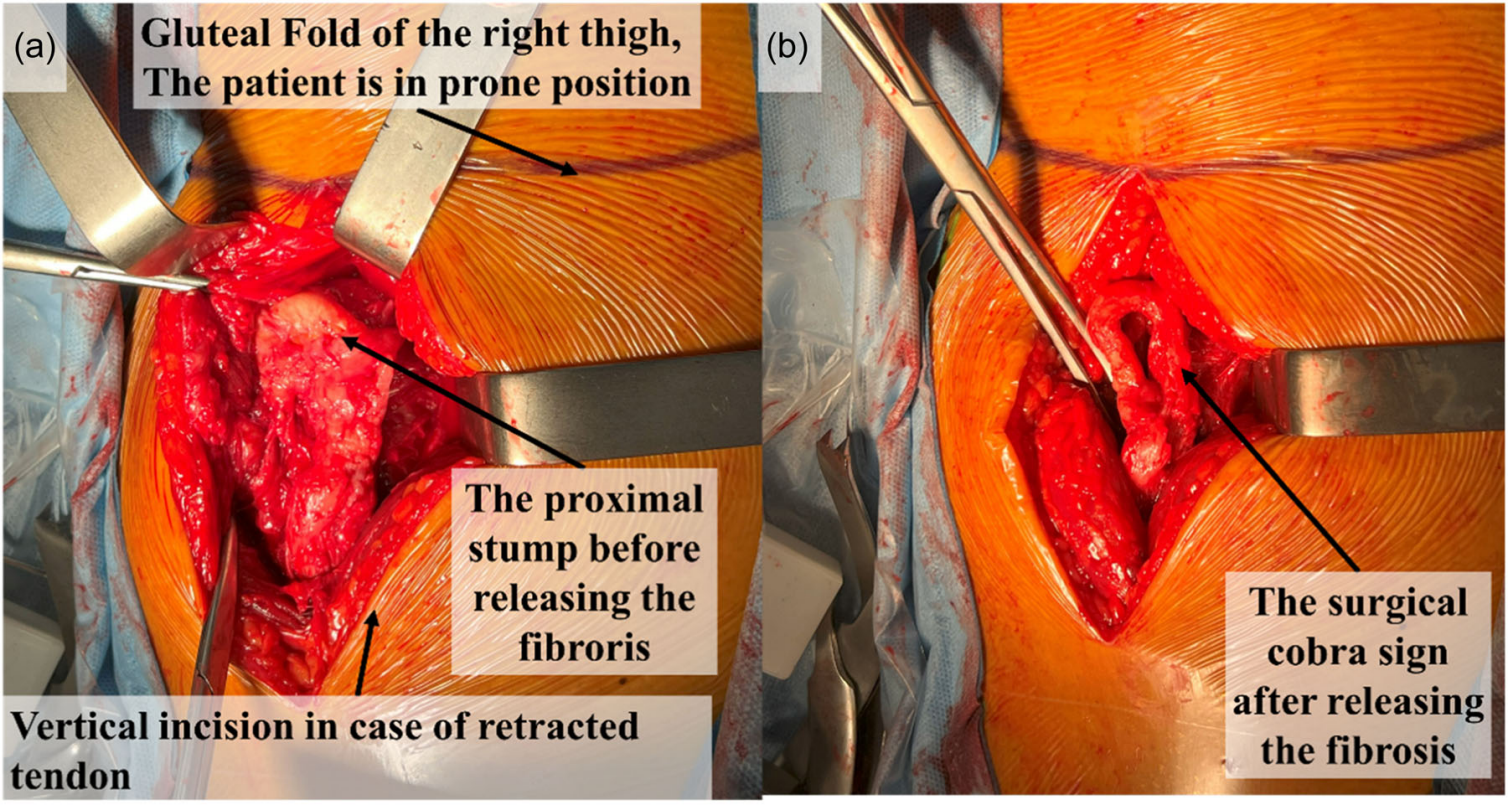
Showing a posterior approach to the right gluteal area in a patient with isolated semimembranosus avulsion placed in the prone position. (a) The proximal stump before releasing the fibrosis. (b) The surgical cobra sign after releasing the fibrosis.

In patients with a positive MRI Cobra sign, care was taken to align the MRI findings with the surgical findings by identifying a surgical Cobra sign, which is characterized by the proximal tendon folding over itself within the stump fibrosis. For patients with a positive surgical Cobra sign, the extremity of the stump was marked with a sterile pen to tag the folding angle. The fibrosis was then opened, the proximal part of the tendon was unfolded, and the folding distance was measured to the nearest millimetre. The true surgical retraction was then determined after unfolding the proximal part (Figure 2, Video S1).

Patients with a negative surgical Cobra sign were considered false positives.

### Outcome measures and evaluation

The primary outcome measure was the characterization of the Cobra sign in patients with proximal hamstring avulsion injury.

This involved describing it by quantifying the folding distance, the largest folding diameter, and the presence of tendon waviness. It also involved the identification of its frequency among different proximal hamstring avulsion injury types, the reliability of this identification, and diagnostic metrics of this sign in identifying each of the proximal hamstring avulsion injury types (sensitivity, specificity, positive and negative predictive values, as well as the positive and negative likelihood ratios). The reference diagnostic standard for calculating the diagnostic metrics was the surgical findings.

The secondary outcome measure was the association of this sign with tendon retraction.

This compared three types of measurement of tendon retraction: the MRI retraction [24], the apparent surgical retraction before unfolding the Cobra sign, and the true surgical retraction after unfolding the sign. The study investigated the odds of having this sign based on MRI retraction and analysed the equation relating MRI retraction to true surgical retraction.

### Data collection

Data were collected prospectively through internet‐based software (Websurvey), which was accessed and filled out by surgeons/fellows. It included patient demographics, injury chronicity, injury type, surgical delay, early MRI findings, and tendon retraction measured from the hamstring footprint to the tendon stump.

### Participants and demographics (Table 1)

**Table 1:** Characteristics of patients included in the study.

Parameter	Total of 81 patients
Age at operation (years)	45.7 ± 13.9
Body mass index	24.3 ± 3.7
Sex	
Males	46 (56.7%)
Females	35 (43.2%)
Affected side	
Left	42 (51.8%)
Right	39 (48.1%)
Type of injury	
Semimembranosus	33 (40.7%)
Conjoint tendon	7 (8.6%)
Complete avulsion	41 (50.6%)
Delay from accident to surgery (in days)	
Acute hamstring injury (<4 weeks)	34 (41.9%)
Chronic hamstring injury (>4 weeks)	46 (59.0%)
Magnetic resonance imaging retraction (cm)	6.6 ± 3.4
Delay to operation (days)	128.1 ± 305.0

Out of 74 patients operated on for proximal hamstring avulsion injury during the first study time frame, 55 patients met the inclusion criteria, of which 41 had complete and 14 partial proximal hamstring avulsion injuries (seven SM, seven conjoint tendons). During the second extended time frame for SM rupture inclusion, 26 patients were included. Finally, a total of 81 patients with proximal hamstring avulsion injuries were included in the study. The mean age at the time of operation was 45.7 years (SD = 13.9). The mean MRI retraction was 6.6 cm, with a mean delay to operation of 128.1 days.

### Statistical analysis

Data were analysed using the *Datatab*® software. Descriptive and inferential analyses were conducted, with the bilateral *T*‐test for independent samples with equal variances being used to measure the strength and direction of the association between the cobra sign and continuous variables such as MRI retraction and surgical delay. The chi‐square test was used to assess the association between the cobra sign and categorical variable such as the need for an allograft for surgical reinsertion and chronicity of injury. Statistical significance was set at a *p*‐value of 0.05. An interrater reliability analysis was performed between the dependent samples of two independent reviewers. For this purpose, the Cohen's *κ* was calculated, which is a measure of the agreement between more than two dependent categorical samples. Because this is a new concept, a formal a priori sample size calculation was not done due to the absence of prior studies describing this concept and hypothesis. Diagnostic metrics, including positive predictive value, negative predictive value, sensitivity, specificity, positive likelihood ratio, and negative likelihood ratio, were calculated to evaluate the diagnostic accuracy of the Cobra sign for each proximal hamstring avulsion injury type. A logistic regression analysis was performed to examine the influence of ‘MRI retraction’ on variable Cobra sign to predict the value ‘Yes’. The coefficient of the variable MRI retraction and its corresponding odd ratio were calculated. A linear regression analysis was performed to examine the influence of the variable ‘MRI retraction’ on the variable ‘Surgical retraction after unfolding the tendon’. The regression coefficient and the regression equation were determined.

## RESULTS

### Characterization of the Cobra sign (Table 2, Figures 1, 2, 3, 4)

**Table 2:** Characterization of the Cobra sign.

Characterization of the Cobra sign	
Type of injury in Cobra positive patients (*N* = 25)	
Semimembranosus	17/25 (68%)
Surgically confirmed	17/17
Conjoint tendon	0/25 (0%)
Complete avulsion	8/25 (32%)
Surgically confirmed	0/8
Reliability (*N* = 25)	
Intraobserver	*κ* = 0.78 (substantial agreement)
Interobserver	*κ* = 0.65 (substantial agreement)
Quantification measurement (*N* = 25)	
Magnetic resonance imaging (MRI) folding‐distance	2.2 ± 0.9
Largest diameter of the folded tendon	1.5 ± 0.6
Waviness	25/25 (100%)
MRI retraction (cm)	
Semimembranosus (*N* = 17)	10.0 ± 3.0
Conjoint tendon (*N* = 0)	N/A
Complete avulsion (*N* = 8)	7 ± 1.6
*p*‐Value	0.014
Diagnostic metric of the MRI Cobra sign in semimembranosus ruptures (*N* = 17/81)	
Sensitivity (Se)	0.51 (95% confidence interval [CI]: 0.345–0.686)
Specificity (Sp)	0.83 (95% CI: 0.728–0.939)
Positive predictive value (PPV)	0.68 (95% CI: 0.497–0.863)
Negative predictive value (NPV)	0.71 (95% CI: 0.596–0.833)
Positive likelihood ratio (PLR)	3.09 (95% CI: 0.884–5.298)
Negative likelihood ratio (NLR)	0.582 (95% CI: 0.364–0.799)

**Figure 3:**
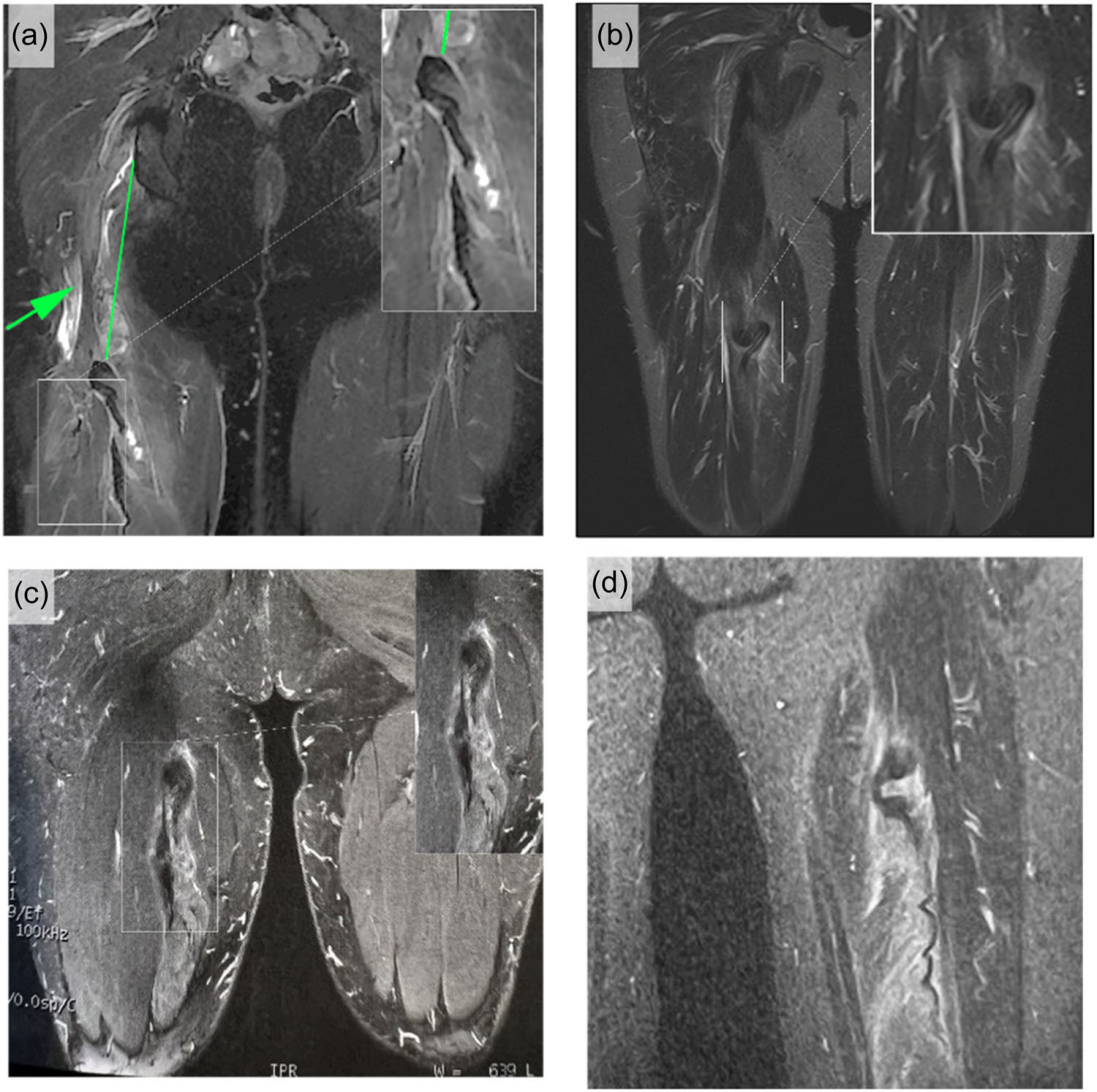
Cobra sign as identified in four patients with isolated semimembranosus rupture. (a) Coronal T2‐weighted MRI of a 50‐year‐old female patient presenting with a domestic injury due to a wide stretch (fall or slip), showing the classic cobra sign with 11 cm retraction. (b) Coronal T2‐weighted MRI of a 52.93‐year‐old male patient presenting with a sports‐related injury due to a wide stretch (fall or slip), showing the classic cobra sign with 13.3 cm retraction. (c) Coronal T2‐weighted MRI of a 48‐year‐old male patient presenting with a sports‐related injury due to a sudden acceleration (rapid start), showing the classic cobra sign with 12 cm retraction. (d) Coronal T2‐weighted MRI of a 55‐year‐old female patient presenting with a sports‐related injury due to a sharp deceleration, showing the classic cobra sign with 9 cm retraction.

**Figure 4:**
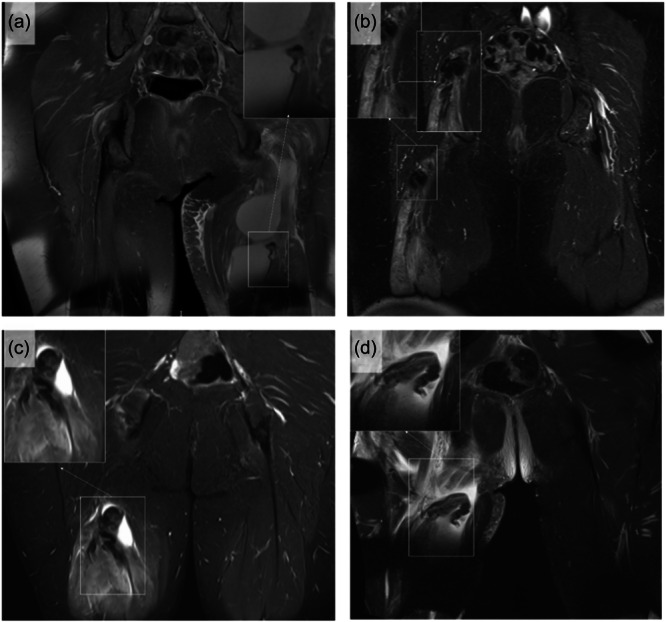
Cobra sign as identified in four patients with complete proximal hamstring avulsion injury. (a) Coronal T2‐weighted MRI of a 53‐year‐old female patient presenting with a sports‐related injury due to a forward fall, showing a complete hamstring avulsion with a Cobra‐like configuration. (b) Coronal T2‐weighted MRI of a 40‐year‐old male patient presenting with a sports‐related injury caused by leg extension and trunk flexion, showing a complete hamstring avulsion with with a Cobra‐like configuration. (c) Coronal T2‐weighted MRI of a 47‐year‐old female patient with a complete hamstring avulsion with a Cobra‐like configuration. (d) Coronal T2‐weighted MRI of a 42‐year‐old male patient presenting showing a complete hamstring avulsion with a Cobra‐like configuration.

The data confirmed that there is a specific configuration of the proximal end of proximal hamstring avulsion injury that appears as a wavy hypointense band with a folded free end that resembles a cobra head surrounded by hyperintense oedema or haematoma (Figures 1, 3, and 4). It was most evident in water‐sensitive sequences (T2‐weighted axial and coronal cuts). In Cobra sign‐positive patients (*n* = 25), most injuries were SM avulsion in 68% (17/25). The descriptive analysis of the Cobra sign features are presented in Table 2. The tendon retraction was higher for SM tendon avulsion (10.0 ± 3.0 cm) than that of the complete tendon (7 ± 1.6 cm) (*p* = 0.014).

*Reliability*: The Cohen's *κ* showed that there was a substantial agreement between both measurement of NL with *κ* = 0.7. Similarly, there was a substantial agreement between both raters (Nicolas Lefèvre and Ahmad Chahal) with *κ* = 0.6.

*Superposability of MRI Cobra sign and surgical Cobra sign:* The Cobra aspect was surgically confirmed exclusively in patients with isolated SM rupture. Therefore, the remaining analysis were reserved to this tendon.

*Diagnostic metrics* (Table 2): The Cobra sign exhibited high specificity of 83.3%, a low sensitivity of 51.5% for isolated SM avulsion.

### Retraction analysis (Table 3)

**Table 3:** Retraction analysis between patients having Cobra signs and those who do not.

	Magnetic resonance imaging (MRI) retraction (cm)	Apparent surgical retraction before unfolding (cm)	Surgical retraction after unfolding the tendon (cm)	*p*‐Value
Retraction analysis in patients with negative Cobra sign (*N* = 56)	5.5 ± 3.12	5.16 ± 2.59	N/A	n.s.
Retraction analysis in patients with positive Cobra sign (*N* = 25)	9.07 ± 2.98	8.24 ± 2.99	7.06 ± 2.47	<0.001
Semimembranosus (*N* = 17)	10.05 ± 3.01	9 ± 3.3	7.91 ± 2.51	<0.001
Complete rupture (*N* = 8)	7 ± 1.6	6.63 ± 1.19	N/A	n.s.

In patients with a positive Cobra sign, MRI retraction significantly overestimated true surgical retraction. Among the 25 patients with a positive sign, MRI retraction was 9.0 cm (±2.9), decreasing to 7.0 cm (±2.4) after surgical unfolding (*p* < 0.001). Specifically, for the 17 SM cases, MRI retraction was 10.0 cm (±3.0), reducing to 7.9 cm ( ± 2.5) postsurgery (*p* < 0.001). No significant difference was found for complete proximal hamstring avulsion injury.

In the logistic regression analysis, MRI retraction significantly influenced the odds of a positive Cobra sign (*χ*²[1] = 19.37, *p* < 0.001). The linear regression analysis applied on patients with isolated SM rupture and positive Cobra sign, indicate that each one‐unit increase in measured MRI Retraction corresponds to a 79% increase in actual surgical retraction, as shown by the equation: Surgical Retraction after unfolding = −0.01 + 0.79 × MRI Retraction. This significant relationship (coefficient 0.79, *t* = 11.1, *p* < 0.001, 95% confidence interval [CI]: 0.64–0.94) suggests an overestimation (by 21%) in tendon retraction when assessed presurgically.

## DISCUSSION

The main finding of this study is that the Cobra sign demonstrated acceptable diagnostic accuracy for isolated SM avulsion, with a high specificity of 83.3%, low sensitivity of 51.5%, and a positive likelihood ratio of 3.0.

### Clinical relevance

The presence of the Cobra sign indicates an overestimated MRI retraction by approximately 21%, as demonstrated by a regression coefficient of 0.79, or by a mean of 2.2 cm (±0.9), as demonstrated by the mean folding distance. The traditional MRI reporting technique does not account for instances with folded tendon, leading to an overestimated measurement of retraction. Furthermore, there is high variability and weakness in the current literature on quantifying tendon retraction, as previously stated [13]. To address this, van der Made et al introduced a standardized method that starts by identifying the ‘ice cream sign’ on axial view as a guide for the determination of proximal full‐thickness free tendon discontinuity and then measuring the shortest distance from the sign to the tendon stump [24]. However, the weak point of this method is the lack of standardization in identifying the tendon stump, which 44% of the radiologists in Six et al.'s study had difficulty determining its location [22]. The proposed adaptation in this study aims to improve the accuracy of tendon retraction calculation by incorporating the concept of apparent retraction. This study builds on the work of van der Made et al. in standardizing MRI reading for proximal hamstring avulsion injury and presents the superiority of providing intra‐operative measurements and validation of the proposed concept of apparent and corrected retraction, which was lacking in their study [24].

This study's adaptation presents two major potential clinical applications. First, it's essential for studies investigating proximal hamstring avulsion injury reparability and the feasibility of its simple repair. Previous research has shown that severely retracted proximal hamstring avulsion injuries are linked to longer operative times and more complex release and difficult repair [12, 25, 26]. Wood et al. published a study of 76 patients with proximal hamstring avulsion injury [26]. They found that complete injury with retraction (average 7 cm, ranging from 2 to 20 cm, in 61%) had worse outcomes compared to those without retraction (21%) and recommended surgery [26]. Understanding that a patient with a 12 cm retraction and a positive Cobra sign might actually have a true surgical retraction closer to 9.38 cm (based on this study's regression equation) can significantly impact surgical planning and expectations.

Second, this adaptation is relevant for studies aiming to establish a retraction threshold for graft augmentation. The trend toward graft augmentation for patients with severely retracted injuries is growing [10, 19]. In 2018, Ebert et al. reported outcomes following proximal hamstring avulsion injury reconstruction using ipsilateral distal hamstring tendon autograft in six patients with chronic ruptures [10]. The authors stated that the decision to use a graft was mainly based on the fact that the retracted proximal hamstring avulsion injury could not be opposed to the ischial tuberosity with the knee flexed to 30°, so a free graft reconstruction was indicated. Assessing various functional and satisfaction metrics over a 24‐month postsurgery period demonstrated good clinical improvement and high levels of patient satisfaction [10].

This study demonstrated that the Cobra sign is more specific for SM injury as evidenced by higher diagnostic metrics, and replication of the surgical Cobra in patients with positive sign. This also supports Lempainen et al's assertion that tears of the biceps femoris, SM, and semitendinosus are not equal and there is a need for a new understanding of individual muscle‐tendon injury in athletes [17]. In 2020, Ayuob et al. published one of the first series of isolated SM ruptures, assessed the efficacy of early surgical intervention on 20 patients with SM, and found a high percentage of satisfaction, return to play, and post‐operative functional scores [5].

### The Cobra sign and its potential mechanism

The potential mechanism that might explain the characteristic ‘Cobra head’ appearance of the avulsed SM tendon is the fact that the SM muscle is the largest muscle in the posterior thigh with the longest proximal tendon [17, 23]. The larger cross‐sectional area results in a greater force it can generate and more stress on its tendon, leading to greater retraction of the SM tendon in the event of rupture [14, 29]. This was evidenced by patients with SM avulsion having higher retraction than those of complete avulsion (10.0 ± 3.0 cm compared to 7 ± 1.6 cm, *p* = 0.014), within the positive Cobra sign group. Conversely, the conjoint tendon of the semitendinosus and biceps femoris muscles has a superficial aponeurotic attachment to the sacrotuberous ligament that may prevent retraction in the case of avulsion or rupture [17, 23]. This high retraction potential combined with the broad proximal insertional aponeurosis of the SM tendon [17, 23], causes it to retract and fold in the form of a cobra. Due to its rarity, there is a scarcity of literature reporting on the mean retraction of SM avulsion, and most available data is retraction for all tendons confound [13, 16, 25]. Wood et al. reported in 2008 a mean retraction of type 5 injury of 7 cm, ranging from 2 to 20 cm [26]. Lefevre et al. reported a mean tendon retraction of 5.8 ± 1.9 in their sample of 34 cases [16]. Wood et al. evaluated the outcomes of 156 proximal hamstring tendon repairs and found the mean tendon retraction for acute repairs was 4.5 cm and for chronic repairs was 4.0 cm (all type confounded, n.s.) [32].

This Cobra sign is evident on coronal T2‐weighted MRI sequences, this sign has not previously been described and characterized in the literature: The classic MRI sign observed in isolated SM avulsion is the complete avulsion of the tendon from its insertion site seen as a bare SM footprint on the upper lateral facet of the ischial tuberosity with adjacent fluid signal along with an intact conjoint tendon [1, 6, 26]. This is best appreciated on axial T2‐weighted MRI sequences and has been previously described as the lateral ‘dropped ice cream sign’ [31]. In the case of a free tendon tear, a small proximal tendon stump is identified at the origin of the tendon attached to the ischial tuberosity [6]. Fluid‐sensitive and T2‐weighted sequences show high‐signal filled defect at the site of tendon disruption consistent with a haematoma that can extend around the sciatic nerve [1, 6, 21]. The avulsed or ruptured tendon is retracted inferiorly, as is the belly of the SM muscle, the tendon usually having a thick, wavy appearance depending on the degree of retraction [1, 6, 21].

The methodology employed in this study has several limitations including sample size and the retrospective nature of the analysis. While the lack of uniformity in the MRI protocols used in this study limits its internal validity, it also reflects the variability in imaging practices in clinical settings, thereby increasing the external validity of the findings. Furthermore, the comparison of MRI retraction was done with surgically measured retraction, which may be influenced by the operator's experience. Indeed, one limitation of this study is the absence of test‐retest reliability measurements to ensure consistency in the surgical retraction measurements.

Despite its limitations, this study's findings provide a new diagnostic marker that can help clinicians better estimate true tendon retraction, improving surgical planning and decision‐making for patients with proximal SM tendon avulsion. Notably, a greater degree of SM retraction does not necessarily indicate an irreparable tendon.

## CONCLUSION

The study demonstrates that the MRI‐identified Cobra sign offers substantial specificity and reasonable sensitivity for isolated SM avulsion. It also establishes a significant correlation between the presence of the Cobra sign and an overestimation of tendon retraction in MRI assessments compared to actual surgical findings. Specifically, the presence of the Cobra sign indicates a systematic overestimation of tendon retraction by approximately 21%. Notably, a greater degree of SM retraction does not necessarily indicate an irreparable tendon.

## AUTHOR CONTRIBUTIONS

Nicolas Lefèvre, Mohamad K. Moussa, Ahmad Chahal, Alain Meyer, Olivier Grimaud, Zeinab M. Khalaf, Ali Alayane and Alexandre Hardy contributed to the conception, design of the study, and interpretation of results. Nicolas Lefèvre, Yoann Bohu, Alexandre Hardy and Ali Alayane contributed to the acquisition of patients and their data. Nicolas Lefèvre, Ahmad Chahal and Zeinab M. Khalaf contributed to review of the database and critical review of data. Nicolas Lefèvre, Ahmad Chahal, Mohamad K. Moussa, Alain Meyer, Olivier Grimaud, Yoann Bohu and Alexandre Hardy contributed to critical review of the manuscript. Mohamad K. Moussa performed the statistical analysis and drafting of tables and figures. Nicolas Lefèvre and Mohamad K. Moussa contributed to the original creation and implementation of clinical databases. Mohamad K. Moussa acts as the guarantor of the study and performed the drafting of manuscript.

## CONFLICT OF INTEREST STATEMENT

Nicolas Lefèvre: Consultant for Websurvey Society, Paris France. Ali Alayane: Consultant for Arthrex and Depuy. The remaining authors declare no conflicts of interest.

## ETHICS STATEMENT

This study involves human participants and was approved by ethics committee (CPP IDF VI). Participants gave informed consent to participate in the study/collection of data before taking part. ClinicalTrials.gov ID: NCT02906865 Patients and/or the public were not involved in the design, or conduct, or reporting, or dissemination plans of this research. Though feedback about the collection of information for the database is encouraged.

## Supporting information

The video demonstrates a posterior view in a patient positioned prone with an isolated semimembranosus avulsion. After releasing the fibrosis, the elasticity of the surgical cobra sign is observed.

## Data Availability

Data are available upon reasonable request.

## References

[1] Askling, C.M. , Koulouris, G. , Saartok, T. , Werner, S. & Best, T.M. (2013) Total proximal hamstring ruptures: clinical and MRI aspects including guidelines for postoperative rehabilitation. Knee Surgery, Sports Traumatology, Arthroscopy, 21(3), 515–533. Available from: 10.1007/s00167-012-2311-0 23229384

[2] Askling, C.M. , Malliaropoulos, N. & Karlsson, J. (2012) High‐speed running type or stretching‐type of hamstring injuries makes a difference to treatment and prognosis. British Journal of Sports Medicine, 46(2), 86–87. Available from: 10.1136/bjsports-2011-090534 22171341

[3] Askling, C.M. , Tengvar, M. , Saartok, T. & Thorstensson, A. (2007) Acute first‐time hamstring strains during high‐speed running: a longitudinal study including clinical and magnetic resonance imaging findings. The American Journal of Sports Medicine, 35(2), 197–206. Available from: 10.1177/0363546506294679 17170160

[4] Askling, C.M. , Tengvar, M. , Saartok, T. & Thorstensson, A. (2007) Acute first‐time hamstring strains during slow‐speed stretching: clinical, magnetic resonance imaging, and recovery characteristics. The American Journal of Sports Medicine, 35(10), 1716–1724. Available from: 10.1177/0363546507303563 17567821

[5] Ayuob, A. , Kayani, B. & Haddad, F.S. (2020) Acute surgical repair of complete, nonavulsion proximal semimembranosus injuries in professional athletes. The American Journal of Sports Medicine, 48(9), 2170–2177. Available from: 10.1177/0363546520934467 32667273

[6] Bencardino, J.T. & Mellado, J.M. (2005) Hamstring injuries of the hip. Magnetic Resonance Imaging Clinics of North America, 13(4), 677–690. Available from: 10.1016/j.mric.2005.08.002 16275576

[7] Bertiche, P. , Mohtadi, N. , Chan, D. & Hölmich, P. (2021) Proximal hamstring tendon avulsion: state of the art. Journal of ISAKOS, 6(4), 237–246. Available from: 10.1136/jisakos-2019-000420 34272300

[8] Bloom, D.A. , Gyftopoulos, S. , Alaia, M.J. , Youm, T. , Campbell, K.A. & Alaia, E.F. (2023) Variability of MRI reporting in proximal hamstring avulsion injuries: are musculoskeletal radiologists and orthopedic surgeons utilizing similar landmarks? Clinical Imaging, 93, 46–51. Available from: 10.1016/j.clinimag.2022.09.001 36375363

[9] Bodendorfer, B.M. , Curley, A.J. , Kotler, J.A. , Ryan, J.M. , Jejurikar, N.S. , Kumar, A. et al. (2018) Outcomes after operative and nonoperative treatment of proximal hamstring avulsions: a systematic review and meta‐analysis. The American Journal of Sports Medicine, 46(11), 2798–2808. Available from: 10.1177/0363546517732526 29016194

[10] Ebert, J.R. , Gormack, N. & Annear, P.T. (2019) Reconstruction of chronic proximal hamstring avulsion injuries using ipsilateral distal hamstring tendons results in good clinical outcomes and patient satisfaction. Knee Surgery, Sports Traumatology, Arthroscopy, 27(9), 2958–2966. Available from: 10.1007/s00167-018-5310-y 30470850

[11] Greenky, M. & Cohen, S.B. (2017) Magnetic resonance imaging for assessing hamstring injuries: clinical benefits and pitfalls—a review of the current literature. Open Access Journal of Sports Medicine, 8, 167–170. Available from: 10.2147/OAJSM.S113007 28761382 PMC5522675

[12] Hillier‐Smith, R. & Paton, B. (2022) Outcomes following surgical management of proximal hamstring tendon avulsions: a systematic review and meta‐analysis. Bone & Joint Open, 3(5), 415–422. Available from: 10.1302/2633-1462.35.BJO-2021-0196.R1 35549447 PMC9134830

[13] Jokela, A. , Stenroos, A. , Kosola, J. , Valle, X. & Lempainen, L. (2022) A systematic review of surgical intervention in the treatment of hamstring tendon ruptures: current evidence on the impact on patient outcomes. Annals of Medicine, 54(1), 978–988. Available from: 10.1080/07853890.2022.2059560 35416097 PMC9009934

[14] Kellis, E. (1998) Quantification of quadriceps and hamstring antagonist activity. Sports Medicine, 25(1), 37–62. Available from: 10.2165/00007256-199825010-00004 9458526

[15] Koulouris, G. & Connell, D. (2003) Evaluation of the hamstring muscle complex following acute injury. Skeletal Radiology, 32(10), 582–589. Available from: 10.1007/s00256-003-0674-5 12942206

[16] Lefevre, N. , Bohu, Y. , Naouri, J.F. , Klouche, S. & Herman, S. (2013) Returning to sports after surgical repair of acute proximal hamstring ruptures. Knee Surgery, Sports Traumatology, Arthroscopy, 21(3), 534–539. Available from: 10.1007/s00167-012-2204-2 22972314

[17] Lefèvre, N. , Coughlan, A. , Valentin, E. , Sezer, H.B. , Bohu, Y. , Moussa, M.K. et al. (2024) Magnetic resonance imaging should be the first‐line imaging modality for investigating suspected proximal hamstring avulsion injuries. Knee Surgery, Sports Traumatology, Arthroscopy, 32, 1862–1870. Available from: 10.1002/ksa.12258 38769849

[18] Lefèvre, N. , Freiha, K. , Moussa, M.K. , Valentin, E. , Bohu, Y. , Meyer, A. et al. (2024) Risk factors for rerupture after proximal hamstring avulsion injury including the optimal timing for surgery. The American Journal of Sports Medicine, 52, 1173–1182. Available from: 10.1177/03635465241233734 38482843 PMC10986149

[19] Lefevre, N. , Kassab Hassan, S. , Valentin, E. , Bohu, Y. , Gerometta, A. , Meyer, A. et al. (2024) Validation of the Parisian Hamstring Avulsion Score (PHAS) in the evaluation and follow‐up of patients operated for proximal hamstring avulsion. The American Journal of Sports Medicine, 52, 1014–1021. Available from: 10.1177/03635465241227434 38353118 PMC10943598

[20] Lefèvre, N. , Moussa, M.K. , Valentin, E. , Meyer, A. , Bohu, Y. , Gerometta, A. et al. (2024) Predictors of early return to sport after surgical repair of proximal hamstring complex injuries in professional athletes: a prospective study. The American Journal of Sports Medicine, 52(4), 1005–1013. Available from: 10.1177/03635465231225486 38353018 PMC10943609

[21] Lempainen, L. , Kosola, J. , Pruna, R. , Sinikumpu, J.‐J. , Valle, X. , Heinonen, O. et al. (2021) Tears of biceps femoris, semimembranosus, and semitendinosus are not equal—a new individual muscle‐tendon concept in athletes. Scandinavian Journal of Surgery, 110(4), 483–491. Available from: 10.1177/1457496920984274 33612019 PMC8688976

[22] Lempainen, L. , Sarimo, J. & Orava, S. (2007) Recurrent and chronic complete ruptures of the proximal origin of the hamstring muscles repaired with fascia lata autograft augmentation. Arthroscopy: The Journal of Arthroscopic & Related Surgery, 23(4), 441.e1–441.e5. Available from: 10.1016/j.arthro.2006.07.044 17418340

[23] Looney, A.M. , Day, H.K. , Comfort, S.M. , Donaldson, S.T. & Cohen, S.B. (2023) Proximal hamstring ruptures: treatment, rehabilitation, and return to play. Current Reviews in Musculoskeletal Medicine, 16(3), 103–113. Available from: 10.1007/s12178-023-09821-7 36757628 PMC9943812

[24] Nishimura, H. , Yamaura, K. , Stetzelberger, V.M. , Garcia, A.R. , Hollenbeck, J.F.M. , Brown, J.R. et al. (2023) Biomechanical comparison of proximal hamstring reconstruction using distal hamstring graft versus fascia lata graft for treatment of chronic hamstring injury. The American Journal of Sports Medicine, 51(14), 3756–3763. Available from: 10.1177/03635465231206464 37975438

[25] Piposar, J.R. , Vinod, A.V. , Olsen, J.R. , Lacerte, E. & Miller, S.L. (2017) High‐grade partial and retracted (<2 cm) proximal hamstring ruptures: nonsurgical treatment revisited. Orthopaedic Journal of Sports Medicine, 5(2), 232596711769250. 10.1177/2325967117692507 PMC5347437 28321428

[26] Rubin, D.A. (2012) Imaging diagnosis and prognostication of hamstring injuries. American Journal of Roentgenology, 199(3), 525–533. Available from: 10.2214/AJR.12.8784 22915392

[27] Shambaugh, B.C. , Olsen, J.R. , Lacerte, E. , Kellum, E. & Miller, S.L. (2017) A Comparison of nonoperative and operative treatment of complete proximal hamstring ruptures. Orthopaedic Journal of Sports Medicine, 5(11), 232596711773855. Available from: 10.1177/2325967117738551 PMC5697601 29201925

[28] Six, W.R. , Buckens, C.F. , Tol, J.L. , Smithuis, F.F. , Maas, M. , Kerkhoffs, G.M. et al. (2020) Reliability of MRI in acute full‐thickness proximal hamstring tendon avulsion in clinical practice. International Journal of Sports Medicine, 42(6), 537–543. Available from: 10.1055/a-1306-0618 33321521

[29] Stępień, K. , Śmigielski, R. , Mouton, C. , Ciszek, B. , Engelhardt, M. & Seil, R. (2019) Anatomy of proximal attachment, course, and innervation of hamstring muscles: a pictorial essay. Knee Surgery, Sports Traumatology, Arthroscopy, 27(3), 673–684. Available from: 10.1007/s00167-018-5265-z 30374579

[30] van der Made, A.D. , Almusa, E. , Whiteley, R. , Hamilton, B. , Eirale, C. , van Hellemondt, F. et al. (2021) Intramuscular tendon involvement on MRI has limited value for predicting time to return to play following acute hamstring injury. British Journal of Sports Medicine, 52(2), 83–88. 10.1136/bjsports-2017-097659 28903949

[31] van der Made, A.D. , Smithuis, F.F. , Buckens, C.F. , Tol, J.L. , Six, W.R. , Lauf, K. et al. (2021) Good interrater reliability for standardized MRI assessment of tendon discontinuity and tendon retraction in acute proximal full‐thickness hamstring tendon injury. The American Journal of Sports Medicine, 49(9), 2475–2481. Available from: 10.1177/03635465211021612 34166119 PMC8283186

[32] Wood, D. , French, S.R. , Munir, S. & Kaila, R. (2020) The surgical repair of proximal hamstring avulsions: does the timing of surgery or injury classification influence long‐term patient outcomes? The Bone & Joint Journal, 102–B(10), 1419–1427. Available from: 10.1302/0301-620X.102B10.BJJ-2019-1112.R1 32993341

